# Delayed Presentation During COVID-19 Pandemic Leading to Post-Myocardial Infarction Ventricular Septal Defect

**DOI:** 10.7759/cureus.15945

**Published:** 2021-06-26

**Authors:** Akshaya Gadre, VeeraPavan Kotaru, Aditya Mehta, Dilpat Kumar, Venumadhav Rayasam

**Affiliations:** 1 Internal Medicine, Western Michigan University Homer Stryker M.D. School of Medicine, Kalamazoo, USA; 2 Cardiology, Ascension Borgess Hospital, Kalamazoo, USA

**Keywords:** ventricular septal defect (vsd), post mi vsd, covid, post mi complications, vsd

## Abstract

Post-myocardial infarction ventricular septal defect (post-MI VSD) is a rare complication of ST-elevation myocardial infarction (STEMI) with an incidence of <1% in early revascularization era. Here we present the case of a 66-year-old woman with post-MI VSD owing to delay in her presentation in the current pandemic. Patient presented with worsening back pain and chest pain with confusion, and an EKG positive for inferior wall STEMI. She underwent emergent percutaneous intervention with placement of drug-eluting stent in her right coronary artery. She developed worsening heart failure and new-onset heart murmur and was found to have a VSD on a transthoracic echo. Because of her poor prognosis, family decided to pursue comfort care and patient unfortunately passed.

Delay in seeking health care during the pandemic, as seen in our patient, is multifactorial including fear of contracting infection, decreased emergency medical services members, and concerns for overburdening healthcare systems. Lack of standardized in-hospital approach to emergencies while ensuring adequate protection from infection to healthcare workers, especially during the initial phase of the pandemic, led to increased door-to-balloon times in addition to the increased time to first medical contact. The importance of media outreach ensuring availability of health care in emergencies, changing emergency response algorithms to ensure safety of patients and healthcare providers, and including thrombolytic therapy where there is a delay due to stringent screening or delayed COVID-19 testing can be used to prevent worsening complications following STEMI.

## Introduction

Post-myocardial infarction ventricular septal defect (post-MI VSD) is a rare but a fatal complication of ST-elevation myocardial infarction (STEMI) [1]. The incidence of post-MI VSD has fallen further since early revascularization, where a door-to-balloon time of 90 minutes is the standard of care. Here we present the case of a 66-year-old woman with a post-MI VSD whose delayed presentation was caused by change in healthcare-seeking behavior during the COVID-19 pandemic. 

Overall STEMI incidences appear to have dropped during the lockdown period due to decrease in environmental exposures [2]. In spite of that, there may be a rise in post-MI complications due to changing patient behavior and changed in-hospital management of STEMI, especially in the early phase of the pandemic [2,3]. This has led to some places observing a higher troponin level at the time of admission, increased in-hospital complications, and lower left ventricular ejection fraction on admission [4].

## Case presentation

**Figure 1:**
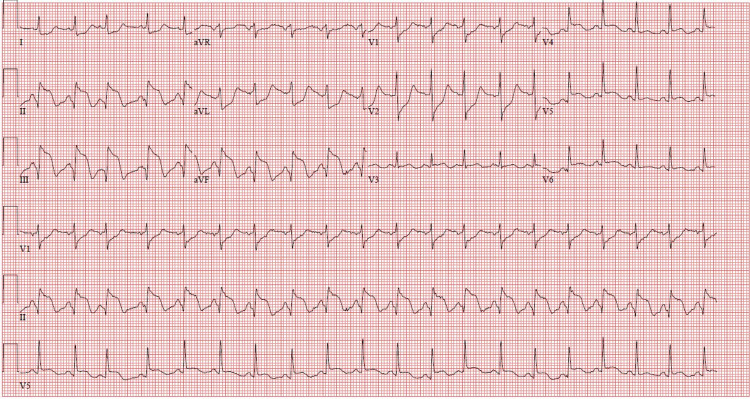
EKG: ST-elevation myocardial infarction in II, III, and aVF with reciprocal changes in V1, V2, and aVL

**Figure 2:**
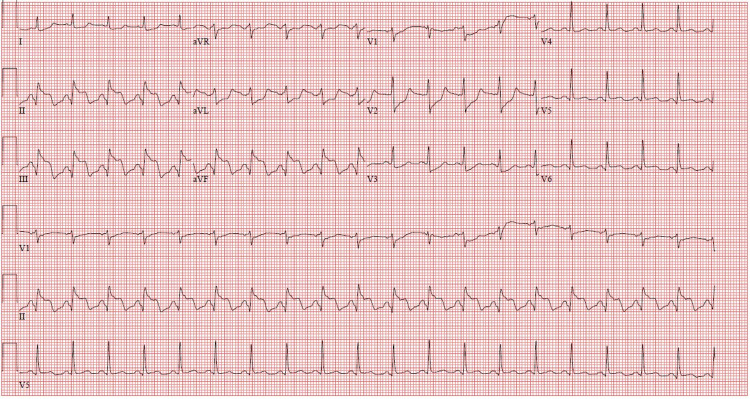
EKG after stent placement. Slight improvement in ST elevation in leads II, III, and aVF

**Figure 3:**
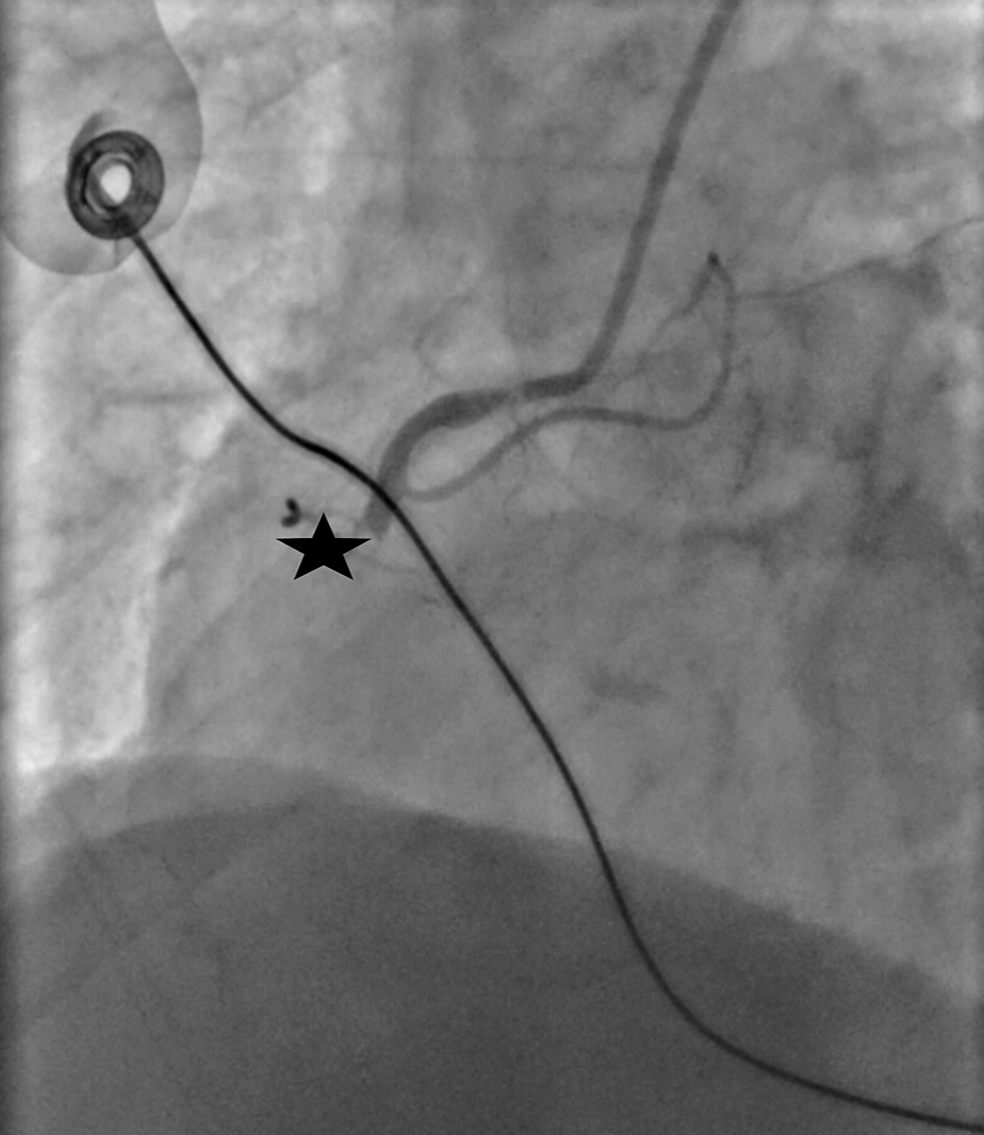
Complete right coronary artery occlusion

**Figure 4:**
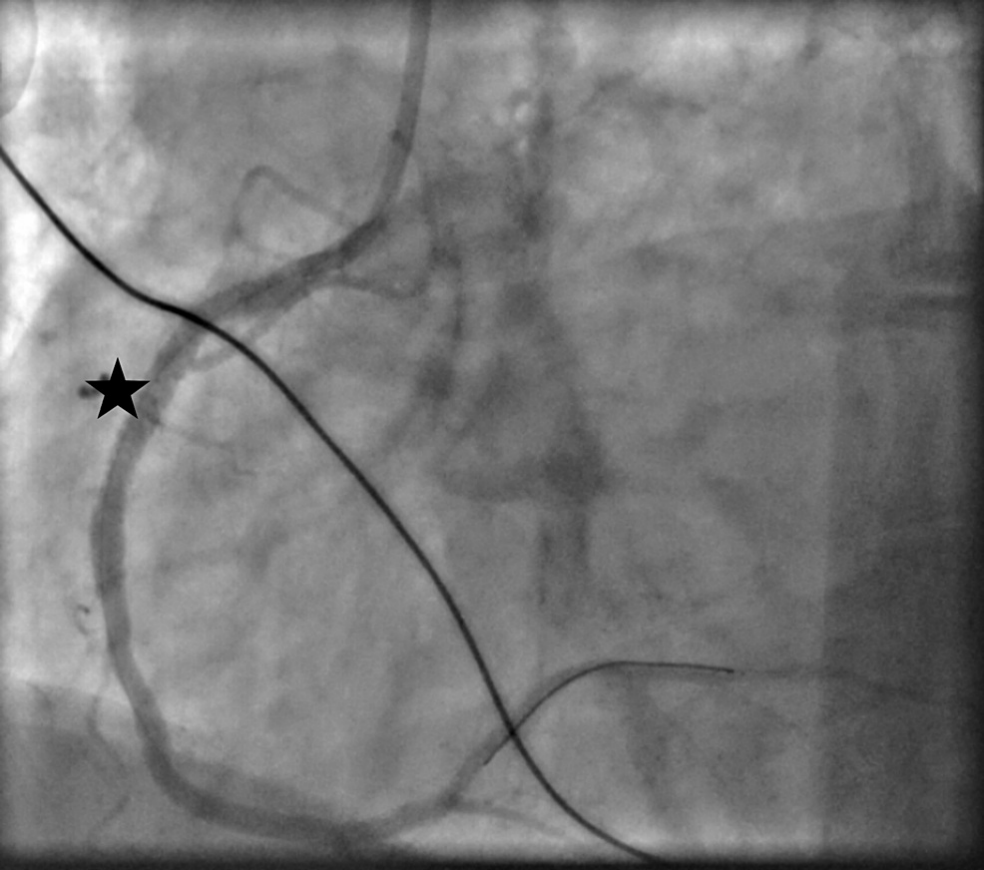
Right coronary artery after ballooning

A 66-year-old woman with a history of chronic obstructive pulmonary disease, chronic back pain, and hypercholesterolemia presented with worsening back pain of two days' duration. She avoided seeking care due to the COVID-19 pandemic and attributed her symptoms to her chronic problems. Emergency medical services (EMS) brought her to the hospital with concerns of STEMI. She received two nitroglycerin and an aspirin en route. On arrival, she was afebrile but tachycardic and in respiratory distress, with a respiratory rate of 30-34 breaths/minute, and oxygen saturation of 98% on room air. She was hypotensive to 56/44 mmHg with confusion. An EKG revealed sinus tachycardia with a ventricular rate of 117 and signs consistent with an inferior wall STEMI, including ST elevation in leads II, III, and aVF (Figure 1). She was given fluids for presumed involvement of right ventricle, which was thought to be contributing to her hypotension. She underwent emergent cardiac catheterization. A 100% occlusion of the proximal right coronary artery was appropriately stented (Figures 2, 3, 4). No VSD was noted during the procedure, and an ejection fraction of 30-35% was recorded.

**Figure 5:**
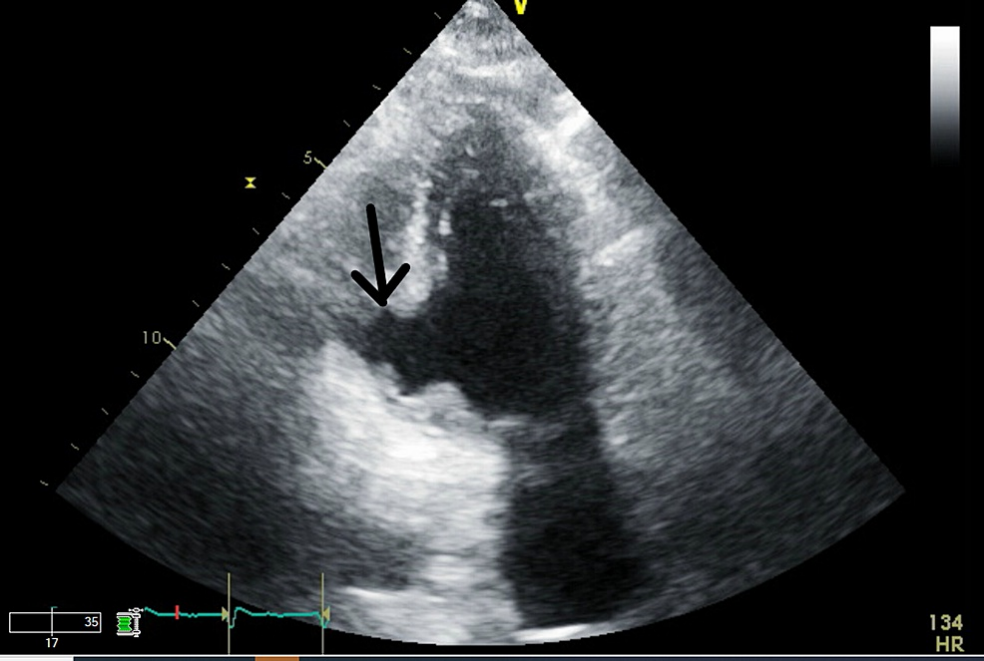
Ventricular septal defect

**Figure 6:**
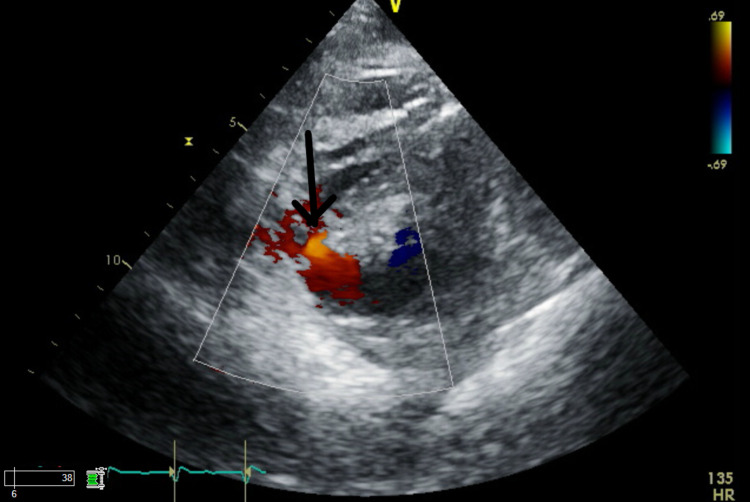
Color Doppler mode showing flow across ventricular septal defect

**Figure 7:**
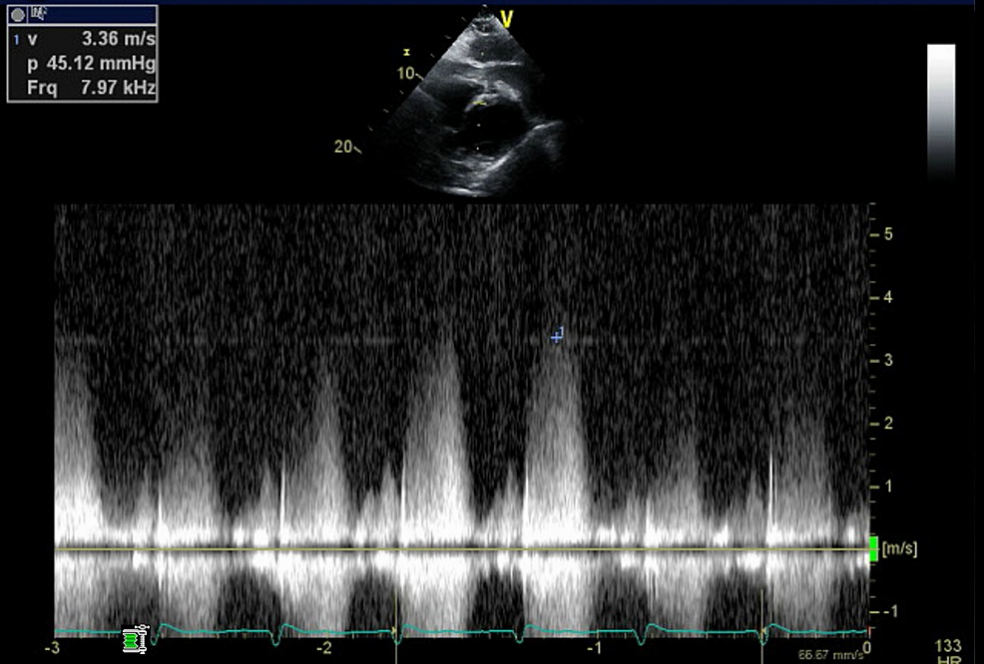
Doppler waveforms measure velocity and pressure gradient across ventricular septal defect

She was admitted to the intensive care unit with respiratory and circulatory failure. The following day, she became increasingly acidotic with lactate levels rising from 7.1 to 12.8 mmol/L. She was also found to have a new pan-systolic murmur, warranting an emergency transthoracic echocardiogram that revealed a VSD (Figures 5, 6, 7). The patient was too unstable for surgical intervention. There were discussions with family regarding the placement of a left ventricular assist device or extracorporeal membrane oxygenation. However, the family opted for comfort care.

## Discussion

Post-MI VSD is the rarest but deadliest complication occurring three to five days following an STEMI. Its incidence decreased to <1% in the early revascularization era [1]. The mechanism is thought to involve the neutrophilic release of lytic enzymes in the coagulation necrosis zone. The fact that the area of absolute infarction is minimal to nil depending on the time from occlusion to reperfusion explains why the incidence has plummeted. Delayed presentations during the pandemic, and delayed interventions due to ever-changing protocols to keep patients and healthcare workers safe, may have contributed to a surge in post-MI complications, as seen in our patient [2,3].

Change in healthcare-seeking behavior seems to have arisen from contamination obsession (a form of obsessive-compulsive disorder focused on hand-washing) exacerbated by the pandemic, worry of being a burden on the healthcare system due to widespread media coverage of an overstretched medical system, and a decrease in EMS workers [5,6]. A direct association between the increased number of COVID-19 infections and a substantial decrease in STEMI undergoing percutaneous coronary interventions (PCI) and a significantly longer door-to-balloon time was noticed [3]. This appears to be due to strict containment measures leading to stringent screening and use of conservative management in select patients considered high risk for COVID-19 infection. The situation was exacerbated by limited personal protective equipment (PPE) and negative pressure catheterization laboratories (cath labs) in the earlier phase of the pandemic [6]. The change in healthcare-seeking behavior should be addressed by engaging in media conversations about importance of seeking care in emergency situations and increasing the frequency of telehealth [7]. Timely treatment with thrombolytics in situations of high COVID-19 burden is an alternative to PCI [8]. Improvement in COVID-19 test turnaround times, increased availability of PPE, and equipping cath labs with negative pressure should also decrease the in-hospital delay in care.

## Conclusions

With social distancing and patients changing their behavior to avoid contracting COVID-19, delayed presentation of all diseases is bound to increase complications. For STEMI, standardized treatment regimens had been responsible for declining complication rates. Healthcare avoidance for fear of contracting COVID-19, concerns for burdening medical systems, and delay in door-to-balloon times due to stringent screening in hospital may have increased complications. Endorsing importance of seeking health care in emergencies in media, use of thrombolytics as an alternative, and improved access to PPE and negative pressure cath labs can be used to address these problems.
